# European Guidelines on HIV and breastfeeding: “Same, same, but different” ‐ Results from a WAVE survey

**DOI:** 10.1111/hiv.70072

**Published:** 2025-08-25

**Authors:** Amy Keane, Lila Haberl, Inka Aho, Stefania Bernardi, Mariana Mărdărescu, Anna Hermine Markowich, Angelina Namiba, Nneka Nwokolo, Keren Olshtain‐Pops, Annette Haberl, WAVE (Women Against Viruses in Europe), European AIDS Clinical Society

**Affiliations:** ^1^ The Department of Genitourinary Medicine and Infectious Disease St James Hospital Dublin Dublin Ireland; ^2^ Department of Gastroenterology, Hepatology and Infectious Diseases, Düsseldorf University Hospital and Medical Faculty Heinrich Heine University Düsseldorf Germany; ^3^ Specialist in Infectious Diseases and Internal Medicine Helsinki University Hospital Helsinki Finland; ^4^ Paediatric infectious disease specialist, Head of Infectious Disease Simple Unit, “Bambino Gesu” Children's Hospital Rome Italy; ^5^ Consultant in Infectious Diseases, Coordinator of Compartment for Monitoring and Evaluation of HIV/AIDS Data in Romania in National Institute for Infectious Diseases “Prof. Dr. Matei Bals”, National HIV Focal Point‐ ECDC Stockholm; ^6^ Department of Pediatrics, Pediatric Infectious Disease Unit, L. Sacco Hospital University of Milan Milan Italy; ^7^ 4M Network of Mentor Mothers London UK; ^8^ Chelsea and Westminster Hospital and ViiV Healthcare London UK; ^9^ Hadassah University Medical Center Jerusalem Israel; ^10^ Department of Internal Medicine, Infectious Diseases, University Hospital Frankfurt Goethe University Frankfurt am Main Germany

**Keywords:** breastfeeding, Europe, guidelines, HIV, recommendations

## Abstract

**Introduction:**

A notable gap exists in research on HIV and breastfeeding in high‐income settings with continuous access to antiretroviral therapy (ART) and suppressed HIV viral loads. The Women Against Viruses in Europe (WAVE) initiative aimed to consolidate European guidelines on HIV and breastfeeding to better inform medical staff and people living with HIV in the decision‐making process for breastfeeding.

**Methods:**

Representatives from 23 countries were contacted by WAVE to submit their guidelines on HIV and breastfeeding, translated into English. The initial contact was made on 20 April 2023, and the final response was received on 26 May 2023. The WAVE breastfeeding group summarized the guidelines into key topics related to breastfeeding for the purpose of this manuscript.

**Results:**

A total of 19 guidelines from 20 countries were included in the review. While the majority of countries recommend formula feeding as the preferred feeding for infants born to mothers living with HIV, most provide recommendations to support parents who choose to breastfeed if certain criteria are met.

**Conclusion:**

Despite recommendations being based on the same research, there is variation across guidelines. This review consolidates European guidelines, enabling us to learn from each other and pool our experiences to create a robust cohort for further research and guideline development for parents living with HIV and their infants.

## INTRODUCTION

In the era of effective antiretroviral therapy (ART), breastfeeding in people living with HIV has become an increasingly discussed topic. Vertical transmission of HIV can occur during pregnancy, delivery and breastfeeding. However, many interventions have reduced the rates of vertical transmission including antenatal HIV testing, effective maternal ART, caesarean sections and infant post‐exposure prophylaxis (Neo‐PEP) [1]. In high‐resource settings, the estimated rate of vertical HIV transmission is <0.5% [2, 3]. Guidelines around breastfeeding differ across Europe. The World Health Organization (WHO) recommends breastfeeding for at least 12 months for all people who have been on effective ART, and even advises that they may continue breastfeeding for up to 24 months or longer, similar to the general population [4]. In settings where diarrhoea, pneumonia and undernutrition are common causes of infant mortality, breastfeeding is a crucial component of preventing child mortality [5]. However, we know that in many high‐resource settings with abundant access to safe, clean water, formula feeding is recommended for children if the breastfeeding person is living with HIV.

Few studies exist on breastfeeding in people living with HIV in high‐resource settings. Switzerland prospectively monitored a cohort of twenty‐one mother‐infant pairs who breastfed, aiming to evaluate the transfer of antiretroviral drugs via breast milk and found that there were zero transmissions within that cohort [6]. The Integrated Screening Surveillance Outcomes in the UK found no cases of HIV vertical transmission attributed to breastfeeding when the British HIV Association (BHIVA) guidance was followed (105 mother‐infant pairs) [3]. However, most of the available data on breastfeeding and HIV comes from low‐resource settings, where access to safe, clean water may not be guaranteed. The Promoting Maternal Infant Survival Everywhere study (PROMISE study) was conducted in sub‐Saharan Africa and India, and was the largest randomized controlled trial directly comparing maternal ART to prolonged infant antiretroviral prophylaxis during breastfeeding [7]. In the PROMISE study, the risk of HIV transmission through breastfeeding, when the mother was on ART (in the absence of infant antiretroviral prophylaxis), was 0.3% after 6 months and 0.7% after 12 months of breastfeeding. It should be noted that in the PROMISE study the majority of mothers received lopinavir/ritonavir‐based ART and only 41% of study participants achieved a viral load below 400 copies/mL at the time of delivery.

However, despite the low rates of transmission seen in the PROMISE study, we cannot apply ‘Undetectable = Untransmittable’ (U=U) to the breastfeeding situation. Despite the risk being lower when the breastfeeding parent has an undetectable viral load, a small risk of transmission to the infant remains [8]. The DoLPHIN 2 study reported a breastfeeding‐associated HIV transmission to an infant despite optimal parental adherence, and virological suppression in a person taking an efavirenz‐based regimen [9]. Similarly in the PROMISE study, the mothers of two of the infants that tested positive in the maternal ART arm, had HIV viral loads that were not detected or detected at <40 copies/mL at the time the infant tested positive for HIV [10].

Breastfeeding is an important part of parental and infant health, providing benefits to the parent and baby, including nutrition, bonding, transfer of maternal antibodies and reduced rates of diabetes and hypertension. In areas with continuous access to effective ART, good sanitation and regular monitoring for both parent and baby, more and more parents would like to breastfeed, or are choosing to breastfeed [3, 11]. Is it right to recommend against breastfeeding in all situations, when the benefits of breastfeeding are well described and although the risk of vertical transmission through breastfeeding is not zero, available evidence suggests it is low? [7].

Over 20 million women and girls in the world live with HIV, more than half of the global population of people living with HIV [12]. As over 1 million of these women and girls give birth each year, it is important that we make the discussion of breastfeeding a priority [13].

Women Against Viruses in Europe's (WAVE) mission is to promote the welfare of women living with HIV in Europe. WAVE consists of health care providers and community representatives who work together to improve the lives of women living with HIV as well as their infants.

Guidelines across Europe differ in relation to breastfeeding. This study is a follow on from the ‘Guidelines and Practice of Breastfeeding in Women Living with HIV ‐ Results from the European INSURE Survey’ conducted in 2022, that asked country representatives to answer a survey on their countries' guidelines and practices in relation to breastfeeding in 25 European countries [14]. Here we compile and review breastfeeding guidelines from 20 countries to inform people living with HIV who wish to breastfeed and their health care providers. This is the first comparison of breastfeeding guidelines across European countries, aiming to support those without national guidelines. We hope this will encourage further discussion on HIV and breastfeeding in Europe.

## METHODS

Respondents from the ‘Guidelines and Practice of Breastfeeding in Women Living with HIV, results from the European INSURE Survey’ who expressed interest in future research were asked to send the relevant section of their guidelines on HIV and breastfeeding [14]. The WAVE breastfeeding group first contacted respondents on 20 April 2023 and received the last guidelines on 26 May 2023. Out of 23 contacted respondents, two had no guidelines on HIV and breastfeeding, one had no official guidelines, two countries shared guidelines, and one was creating new guidelines, resulting in 18 initial guidelines. Belgium, Sweden and Turkey updated their guidelines during this period which were included in the review. France updated their guidelines in 2024 and were invited during the development of the manuscript to submit their guidelines, leading to a total of 20 countries' guidelines (19 guidelines) included in the manuscript. The guidelines were mainly translated into English by the country representatives, who are often part of the national guidelines committee. In cases where only the native language version was available, a translation programme was used. The results reflecting each countries guidelines were submitted to the country representatives for possible correction. A group of physicians and community representatives analysed the results. Regular meetings were held whereby key topics required in a comprehensive guideline were decided on and guidelines subsequently analysed for information relating to each topic.

## RESULTS

### Summaries

**FIGURE 1:**
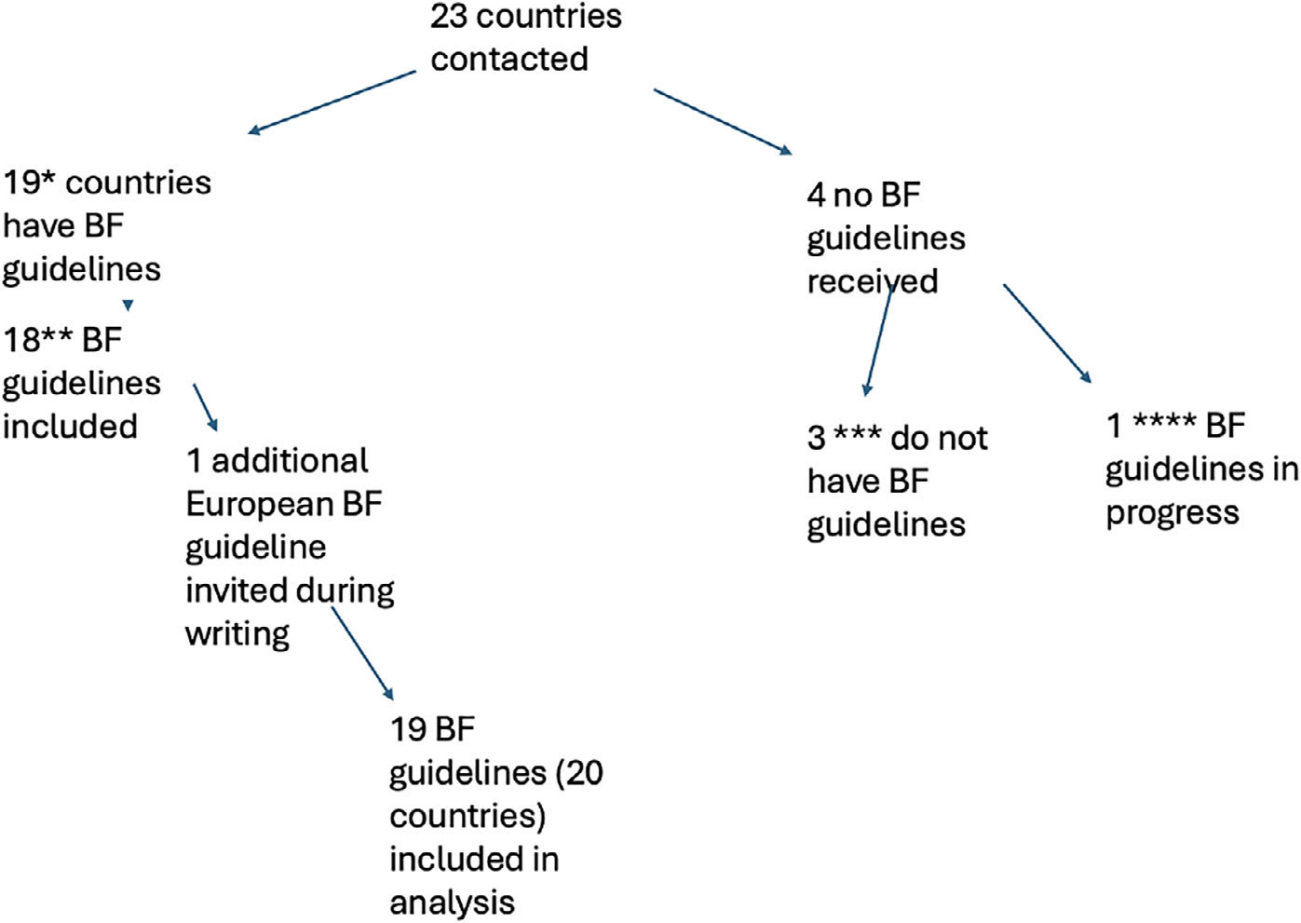
Breastfeeding guidelines received/not available. *Austria, Belgium, Czech Republic, Denmark, Germany, Greece, Ireland, Italy, Latvia, Poland, the Netherlands, Norway, Russia, Spain, Sweden, Switzerland, Turkey, Ukraine and the UK. **Germany and Austria share guidelines. ***Israel, Portugal, Finland. ****Romania.

The following countries provided access to their most recent guidelines which have been summarized below; Austria, Belgium, Czech Republic, Denmark, France, Germany, Greece, Ireland, Italy, Latvia, the Netherlands, Norway, Poland, Russia, Spain, Sweden, Switzerland, Turkey, Ukraine and the United Kingdom. Please see Figure 1 for a flowchart of countries' guidelines received or not available (Table 1).

**TABLE 1:** Summary of information available on breastfeeding countries' guidelines.

	Belgian	Czech Republic	Denmark	France	German/Austrian	Greece	Ireland	Italy	Latvia
Year of publication	2023	2019	2020	2024	2020	2020	2015	2018	2014
Main reccomendation about breastfeeding	Possible (conditions)	Recommend against	Possible (conditions)	Possible (conditions)	Possible (conditions)	Possible (conditions)	Recommend against (only exceptional cases)	Recommend against	Recommend against
Neonatal PEP (in general)	Low risk: no PEP, intermediate+ high risk: PEP	No data	No data	Yes	Low risk: no PEP	No data	Yes	No data	No data
Prolonged PEP because of breastfeeding	Only if parents request it	No data	No data	Yes	No	No	No data	No data	No data
Interval of maternal viral load testing	6 weeks	No data	Yes	4 weeks	d1, d14, d28, M2, M4, M6, then every 2 month	4 weeks	No data	No data	No data
Interval of infant viral load testing	6 weeks	No data	Yes	M1, M3, M6, then every 3 months	d0, d14, d28, month 2, month 6	4 weeks	No data	No data	No data
Stop criterias	Yes	No data	Yes	Yes	Yes	Yes	No data	No data	No data
Benefits of breastfeeding mentioned	No	No	No	No	Yes	No	No	No data	No data
Interdisciplinary team recommended	Yes	No	No	Yes	Yes	Yes	No	No data	No data
Peer support recommended	No	No	No	No data	No	No	No	No data	No data
Follow up of the breastfed infant	Yes	No data	yes	Yes	yes	Yes	No data	No data	No data
Suggested duration of breastfeeding	<6 months	No data	As short as possible	<6 months	No	No	No data	No data	No data
Recommendation about mixed feeding	No	No data	Yes	Yes	Yes	No	No data	No data	No data

	Netherlands	Norway	Poland	Russia	Spain	Sweden	Switzerland	Turkey	Ukraine	UK
Year of publication	2023	2023	2023	2020	2018	2024	2018	2024	2022	2020
Main reccomendation about breastfeeding	Possible (conditions)	Possible (conditions)	Possible (conditions)	Recommend against	Recommend against	Recommend against	Possible (conditions)	Possible (conditions)	Possible (conditions)	Possible (conditions)
Neonatal PEP (in general)	Yes	Yes 4 weeks	No data	Yes	No data	Yes	No (optimal scenario)	Yes	No data	Yes
Prolonged PEP because of breastfeeding	Optional	No	No data	Yes (6 weeks)	No data	No	No	No data	No data	No
Interval of maternal viral load testing	Intensive monitoring	4 weeks	4 weeks	No data	No data	4 weeks	4 weeks (then 2–3 months)	4–8 weeks	4 weeks	4 weeks
Interval of infant viral load testing	4 weeks	4 weeks	4 weeks	No data	No data	4 weeks	month 1, month 2, month 6	4–8 weeks	4 weeks	4 weeks
Stop criterias	No data	Yes	No	No data	No data	Yes	Yes	Yes	Yes	Yes
Benefits of breastfeeding mentioned	No	No	No	No	No	Yes	Yes	No	No	No
Interdisciplinary team recommended	Yes	Yes	Yes	No data	No data	No data	Yes	No	Yes	Yes
Peer support recommended	No	No	No	No data	No data	No data	No data	No	No	Yes
Follow up of the breastfed infant	Yes	Yes	Yes	No data	No data	Yes	Yes	Yes	Yes	Yes
Suggested duration of breastfeeding	<6 months	<6 months	<6 months	No data	No data	<6 months	<6 months	<6 months	No data	As short as possible <6 months
Recommendation about mixed feeding	No data	Yes (not recommended)	No	No data	No data	Yes (not recommended)	No data	Yes	Yes	Yes

While the majority of countries recommend formula feeding primarily, the following is a summary of countries' guidelines for supported breastfeeding in optimal scenarios.

### Criteria for supported breastfeeding

Ten guidelines define criteria which are needed to be met before breastfeeding should be supported (Belgium, France, Germany/Austria, Ireland, Norway, Switzerland, Sweden, Turkey, Ukraine and United Kingdom). All of them (10/10) require a suppressed parental HIV viral load (where defined in the guidelines this was a HIV viral load <50 copies/mL), a good ART adherence history, and regular testing during the breastfeeding period.

### Guidance and support on breastfeeding

Multidisciplinary support (e.g.from paediatricians, midwives, HIV physicians, obstetricians and lactation consultants) is advised in the guidelines from Belgium, Germany/Austria, Greece, Ireland, Norway, Poland, and Switzerland. Belgium, Germany/Austria, and the United Kingdom also make reference to the need for an information leaflet to help parents in decision making.

Few guidelines mention peer support as a resource for parents who choose to breastfeed. The UK guidelines recommend that parents are strongly encouraged to inform partners/families and health care providers (including midwives, health visitors, and GPs) about their HIV status to enable them to obtain advice and support. The Belgium guidelines recommend involving the father in the decisionmaking process if possible.

In Germany, employees from AIDS organizations and other services for people living with HIV have been identified as potential providers of support. Sweden advises an information session on infant feeding which includes information on transmission risk, discussing the parents’ desire to breastfeed, stigma, and feelings of grief/loss if not breastfed.

### Benefits/challenges of breastfeeding

Some guidelines highlight the benefits and risks of breastfeeding. For example, the Ukrainian guidelines recommend counselling on formula feeding and the risk of transmission of HIV through breastfeeding. The UK guidelines highlight the emotional, financial and social costs of not breastfeeding, noting that some parents might forgo their own nutritional needs in order to afford formula for their infant, thus compromising their own health. Some guidelines advise providing free formula to alleviate this burden. Many guidelines mention the risk of transmission of HIV through breastfeeding. Switzerland describes issues with the requirement for intensified monitoring during breastfeeding in the vulnerable post‐partum period, which could lead to impaired adherence and consequential increased viral load. The German/Austrian guidelines address discrimination in cases where the parent is not breastfeeding, such as shared accommodation for migrants.

Both German/Austrian and Swiss guidelines address the benefits of breastfeeding, including nutrients for child development and immune health, as well as maternal benefits like uterine involution, reduced post‐natal depression and breast cancer risks, and a stronger mother–child bond. Breastfeeding may also be considered a simple, easy and free means of providing nutrition to the infant.

## MANAGEMENT AND MONITORING

### Viral load monitoring

Of all guidelines reviewed in the survey, 12 discuss monitoring of the parent and infant during the breastfeeding period. The following eight guidelines: Germany/Austria, France, Greece, Ireland, Norway, Poland, Ukraine, and UK state that follow‐up of the breastfeeding parent with viral load testing should be performed monthly throughout breastfeeding. Swiss guidelines state that testing be performed monthly during the post‐partum period and every 2–3 months thereafter, while the Belgian guidelines recommend viral load monitoring every 6 weeks, with HIV RNA measurement performed on breast milk if available. The Turkish guidelines recommend viral load testing every 1–2 months.

As for the infant, the nine countries' guidelines mentioned above plus the Netherlands recommend monthly viral load testing throughout breastfeeding, while the Swiss guideline suggests less frequent testing as follows: at 1 and 6 months, as for non‐breastfeeding infants, with additional testing at months 2 and/or 4 months to be considered. Swiss guidelines also advise sampling cord blood at birth to confirm the presence or absence of HIV RNA acquired in utero. The Belgian guidelines recommend viral loads every 6 weeks for the mother. Regarding infant testing after discontinuation of breastfeeding, Norway, Poland, Ukraine and the UK all suggest final testing 2 months after weaning, while the Swiss, French and Belgian guidelines suggest 3 months after weaning.

### Mixed feeding

The definition of mixed feeding was not clear across many of the guidelines. In this context, we consider mixed feeding as the introduction of formula or solids before 6 months of age, but it remains unclear if this definition is followed by all guidelines. Ireland, Spain, Sweden and Ukraine highlight that mixed feeding is considered a risk factor for HIV transmission during breastfeeding, with Ukraine describing the highest risk as mixed feeding with solids before 2 months of age. The guidelines from Denmark, Norway, Sweden and Turkey specifically recommend against mixed feeding. The Danish guidelines describe mixed feeding in this scenario as taking breastmilk and solids together, with Ireland also specifying exclusive breastfeeding only and to stop breastfeeding once solids are introduced. The UK guidelines do not recommend breastfeeding together with solids before 6 months of age due to the significantly increased HIV transmission risk, but they do include a list of conditions in which mixing breastfeeding and formula feeding might be supported. These conditions are: establishing breastfeeding, switching from breast milk to formula milk, mastitis, gastroenteritis in the feeding parent or the infant. Sweden also advises that formula can temporarily be used in the case of mastitis, with Norway also specifying that formula can be used in the short term when establishing breastfeeding.

### Infant management – Neonatal post‐exposure prophylaxis (PEP) / Neonatal pre‐exposure prophylaxis (PrEP)

The recommendations regarding the need and the timing of infant PrEP/PEP vary across the guidelines. In Austria, Germany, Greece, the Netherlands, Norway, Switzerland, Turkey and the UK, the decision to breastfeed does not change the recommendation for PrEP/PEP. In case of a mother's wish to breastfeed, the Irish guidelines suggest a discussion with adult and paediatric services, so that maternal therapy and infant prophylaxis can be planned. France recommends extending PEP for the infant for the duration of breastfeeding and for 15 days after its cessation. Sweden advises considering infant PEP if the parent's HIV viral load is measured at >200 copies/mL during breastfeeding (and breastfeeding should be ceased). The other guidelines report no specific PrEP/PEP recommendations during breastfeeding.

### How/when to stop breastfeeding

#### Reasons to stop breastfeeding

Most guidelines specifically advise cessation of breastfeeding if the viral load is above 50 copies/mL. The German/Austrian and Belgian guidelines advise that in the case of a minor viral blip (defined as a viral load of 50–199 copies/mL), a decision to resume breastfeeding can be made on a case‐by‐case basis once the viral load has become undetectable again. Sweden further adds that in the case of a viral blip in the setting of good adherence, breastfeeding should be ceased and the viral load repeated within 1–2 weeks. If the viral load remains >50 copies/mL, then it is recommended to stop breastfeeding.

Additional factors on when to stop breastfeeding include whether the parent has symptoms of mastitis or bleeding nipples. Other reasons documented in the various guidelines include gut infections (gastro‐intestinal symptoms, e.g., diarrhoea) for both mother and baby, and if the baby has mouth infections. The Belgium, Norwegian, French and Danish guidelines recommend using the other breast if healthy in the case of mastitis/cracked nipples. France does not advise stopping breastfeeding in the case of cracked nipples.

#### Duration of breastfeeding

Guidance on the duration of breastfeeding varies, with many countries not addressing this. Some countries make strong recommendations for the length of time that breastfeeding should continue, while others are less stringent. The Danish, Norwegian, Swedish, Turkish and United Kingdom guidelines recommend breastfeeding for a maximum of 6 months, with the UK, Denmark and Norway advising that abrupt weaning from breastfeeding is not necessary. Other countries, such as Ireland, advise that breastfeeding should not be continued once solids are commenced. The German/Austrian guidelines do not limit the duration of breastfeeding.

#### Weaning

Six guidelines discuss weaning to solids. Denmark, Norway, Sweden, Turkey and the United Kingdom recommend weaning to complementary foods after 6 months of age. Germany/Austria do not recommend introducing solids before 4 months of age.

#### Suppression of lactation

Nine guidelines discuss suppression of lactation. Belgium, Greece, Poland, Germany/Austria, Ukraine and the United Kingdom recommend the use of cabergoline to suppress lactation in women living with HIV who are not breastfeeding. Greece states that this is to prevent the potential physical and emotional discomfort associated with breast engorgement and also the risk of covert breastfeeding. Ireland, Norway and Sweden advise providing support to women to suppress lactation.

## DISCUSSION

This study collated guidelines on HIV and breastfeeding from 20 European countries. Most of the guidelines support decisions to breastfeed if certain criteria are met. Some countries, such as Italy and Romania, are currently creating or modifying guidelines which will further support breastfeeding parents, while others, like Finland, follow non‐official guidance. Although there is variation in guidelines, similarities include the criteria to support breastfeeding and recommendations on parental and infant viral load testing, with all recommending close monitoring of the breastfeeding parent and infant. However, diversity exists, such as in the areas of duration of breastfeeding. The guidelines lack clarity on the definition and recommendations of mixed feeding. The differences in guidelines partly stem from limited research in resource‐rich settings with effective ART [15]. Variations in the interpretation of available data have led to differing guideline recommendations, despite being based on the same evidence. It is uncertain how prior research applies to resource‐rich settings, particularly for those with sustained viral loads and continuous access to effective ART. This marks a transitional period for parents and healthcare providers, shifting from a policy of zero breastfeeding in resource‐rich settings to endorsement of breastfeeding under conditions of sustained viral suppression.

Most countries recommend monthly viral load monitoring during breastfeeding for both parent and infant though the optimal interval of monitoring is unclear [1]. Close monitoring is essential for promoting adherence [16] and allowing swift action in the case of virological failure, but it can add stress during the vulnerable post‐partum period [17]. Close monitoring also reflects the providers' unease due to lack of research on breastfeeding and suppressed viral loads. Many countries advise ceasing breastfeeding if the viral load is >50 copies/mL though guidance on management of viral blips is limited.

Some countries recommend stopping breastfeeding by 6 months, based on the PROMISE study, which showed a 0.3% HIV transmission risk after 6 months and 0.7% after twelve months when the parent was on ART. [7]. Notably, the transmission risk at 18 months remained at 0.7%. This raises the question, does the benefit of breastfeeding up to 2 years of age, as recommended by the WHO, outweigh the small additional risk associated with extending breastfeeding beyond 6 months? As mentioned in the introduction, in the PROMISE study the majority of mothers received ART that is not commonly used anymore, and less than half of the study participants achieved a viral load below 400 copies when commencing breastfeeding.

Most guidelines advise to cease breastfeeding in the case of mastitis. Much of the data on mastitis and increased risk of transmission of HIV through breastfeeding comes from the pre‐ART era [18, 19]. It is unclear if transmission risk remains the same in the setting of effective ART [1, 20]. Mastitis, particularly in the first 6 weeks, is common, occurring in 3%–20% of breastfeeding people [21]. Further research is necessary.

The WHO defines mixed feeding as giving an infant under 6 months liquids or solids alongside breastmilk. [5] Many guidelines were unclear whether this referred to combination feeding with formula or early introduction of solids, or both. The United Kingdom BHIVA position statement on HIV and mixed infant feeding primarily recommends exclusive breastfeeding before 6 months of age, but gives examples of when formula can supplement breastfeeding. However, with the caveat that this should only be done in certain situations, as the risk of HIV transmission with mixing breast and formula feeding is unquantifiable with current evidence [22]. The WHO advises that mixing formula and breastfeeding is better than not breastfeeding at all due to the benefits of breastmilk [23]. Few studies have looked at mixed feeding in the context of ART [24]. In one analysis of breastfeeding women living with HIV not on ART, exclusively breastfed babies were compared with those given water‐based drinks and solids. Infants who were primarily breastfed and also given water‐based drinks did not have a higher rate of HIV acquisition versus those who were exclusively breastfed. However, infants who were given solids before 6 months did have a higher rate of HIV acquisition compared with those exclusively breastfed (when the parent was not on ART) [25].

Supporting parents to breastfeed, when clinically appropriate, is important, and this should be reflected in guidelines. Peer‐led programmes, like the 4 M network in the United Kingdom, offer vital support through pregnancy and beyond. [26] Whether breastfeeding or not, this is a challenging time and support is vital [27]. Community representation is important in the creation of guidelines, for example, Nourish‐UK interviews parents living with HIV about infant feeding choices to help inform guidelines [28].

This review summarizes published guidelines on HIV and breastfeeding, aiming to present the data clearly. Due to space limitations, the full text of each guideline is not included; instead, an overview is provided. The guidelines were originally in their native languages and were translated into English for the review. Every effort was made to accurately represent each guideline, with results sent for review by a representative from each country. Although this manuscript only includes guidelines from 20 countries (19 guidelines), and thus does not provide a complete picture of all European guidelines, it does cover nearly half of Europe. The working group, composed of physicians and community representatives from seven countries, ensured diverse representation through the WAVE network, fostering collaboration.

Based on our review of the guidelines, we have made recommendations for what should be included in a comprehensive guideline on HIV and breastfeeding (Table 2).

**TABLE 2:** Topics to be included and discussed in a comprehensive guideline on HIV and breastfeeding.

	Headings	Sub‐headings
1.	Criteria for breastfeeding	e.g., duration of suppressed maternal viral load
2.	Available guidance and support	Interdisciplinary setting/peer support
3.	Monitoring during breastfeeding	Infant/maternal viral load monitoring Including premature infants
4.	Infant feeding	Mixed feeding Weaning process
5.	Duration of breastfeeding	Is there any time limit at all and if so how long is breastfeeding recommended
6.	Time of decision making	
7.	Suppression of lactation	
8.	Stop criteria	
9.	Infant PEP/PrEP	Including duration and what to do in the case of viral rebound in the breastfeeding parent
10.	Advice in certain situations	e.g., mastitis, viral blips

With limited research in the era of effective ART in resource‐rich settings, we must pool our experience of breastfeeding in parents living with HIV in Europe to help inform future, comprehensive and relevant guidelines. Sharing knowledge is essential to ensure that parents and their infants receive optimal care, while also equipping physicians with robust, evidence‐based guidelines to support them. We urge everyone working in the field of HIV, breastfeeding, antenatal medicine and beyond to equip themselves with the knowledge to aid those living with HIV who wish to breastfeed. We want to call to action clinicians and researchers, and encourage them to increase research output in high‐income settings, so that we can bridge data gaps and provide the best quality evidence‐based medicine to parents and their infants.

## AUTHOR CONTRIBUTIONS

The authors confirm their contributions to the paper as follows: Study conception and design: AH, AK, LH; Data collection: AK, LH; Analysis and interpretation of results: IA, AH, LH, NN, AM, MM, AM, AK, SB, KOP; Draft manuscript preparation: IA, AH, LH, NN, AM, MM, AM, AK, SB, KOP; All authors reviewed the results and approved the final version of the manuscript.

## CONFLICT OF INTEREST STATEMENT

IA (Inka Aho) received fees from Gilead, GSK, Merck and support for congress participation from Gilead. AH (Annette Haberl) received lecture fees from Gilead Sciences, MSD, and ViiV Healthcare and also support for congress participation from Gilead Sciences. LH (Lila Haberl) received lecture fees from ViiV. NN (Nneka Nwokolo) is a part‐time employee of ViiV Healthcare. AM (Anna Hermine Markowich), MM (Mariana Mărdărescu), AM (Angelina Namiba), AK (Amy Keane), SB (Stefania Bernardi), KOP (Keren Olshtain‐Pops): declare no conflict of interest.

## Data Availability

The data that support the findings of this study are available from the corresponding author upon reasonable request.
